# P-2190. Gender Differences in Hepatitis C Treatment in the New York City Jail System, 2019-2023

**DOI:** 10.1093/ofid/ofae631.2344

**Published:** 2025-01-29

**Authors:** Chibuzo U Enemchukwu, Raphael Simonson, Batya Koenigsberg, Natalia Gonzalez Varela, Jacqueline LaPene, Richard Figueroa, Janet Wiersema

**Affiliations:** New York City Health+Hospitals Correctional Health Services, New York, New York; New York City Health+Hospitals Correctional Health Services, New York, New York; New York City Health+Hospitals Correctional Health Services, New York, New York; New York City Health+Hospitals Correctional Health Services, New York, New York; New York City Health+Hospitals Correctional Health Services, New York, New York; New York City Health+Hospitals Correctional Health Services, New York, New York; New York City Health+Hospitals Correctional Health Services, New York, New York

## Abstract

**Background:**

Little is known about Hepatitis C (HCV) care outcomes among women experiencing incarceration. Women make up only 7% of the New York City (NYC) jail population, but represent 18% of individuals diagnosed with active HCV. This study examines progress through the HCV care cascade among women and men in the NYC jail system and describes potential gender-related factors in cascade outcomes.

**Methods:**

Persons admitted to the NYC jail system and who completed a medical intake between October 2019 and December 2023 were included in this retrospective cohort analysis. We constructed a care cascade describing rates of universal opt-out HCV screening, confirmatory testing, diagnosis, linkage to jail-based clinical HCV care, and jail-based HCV treatment among the jail population stratified by documented gender. Demographic characteristics and medical history were also examined and stratified by documented gender.

**Results:**

71,130 persons were included in our analysis, of whom 5,085 (7.1%) were women and 66,045 (92.9%) were men. Compared to men, women were screened at higher rates (88.7% vs. 28.1%; p< 0.001), underwent confirmatory testing at lower rates (9.6% vs 11.2%; p=0.002), and had similar rates of active HCV (51.2% vs 47.8%; p=0.22). Women with active HCV were seen by an HCV clinician at similar rates as men (55.4% vs. 59.6%; p=0.32) but initiated HCV treatment at nearly half the rate of men (15.3% vs. 30.3%; p< 0.001). Additionally, women in the NYC jails had significantly higher rates of homelessness (23.5% vs. 19.5%), HIV (3.7% vs. 2.7%), mental health needs (53.7% vs. 30.8%), and history of select substance use disorders (58.6% vs. 45.8%) when compared to men (p< 0.001).

**Conclusion:**

Large HCV treatment gaps exist between women and men experiencing incarceration. Disproportionally higher rates of comorbid medical, mental health, and substance use disorders and more re-entry support needs likely contribute to treatment disparities. Gender-specific strategies centered on the needs of women with justice involvement are needed to enhance the equitable uptake of jail-based HCV treatment. Enhancing treatment access for all persons with justice involvement is critical for HCV elimination efforts.

**Disclosures:**

All Authors: No reported disclosures

